# Skeletal Muscle Tissue Engineering: Biomaterials-Based Strategies for the Treatment of Volumetric Muscle Loss

**DOI:** 10.3390/bioengineering7030085

**Published:** 2020-07-31

**Authors:** Meagan E. Carnes, George D. Pins

**Affiliations:** Department of Biomedical Engineering, Worcester Polytechnic Institute, 100 Institute Rd., Worcester, MA 01609, USA; mecarnes@wpi.edu

**Keywords:** biomaterials, tissue engineering, volumetric muscle loss, skeletal muscle regeneration

## Abstract

Millions of Americans suffer from skeletal muscle injuries annually that can result in volumetric muscle loss (VML), where extensive musculoskeletal damage and tissue loss result in permanent functional deficits. In the case of small-scale injury skeletal muscle is capable of endogenous regeneration through activation of resident satellite cells (SCs). However, this is greatly reduced in VML injuries, which remove native biophysical and biochemical signaling cues and hinder the damaged tissue’s ability to direct regeneration. The current clinical treatment for VML is autologous tissue transfer, but graft failure and scar tissue formation leave patients with limited functional recovery. Tissue engineering of instructive biomaterial scaffolds offers a promising approach for treating VML injuries. Herein, we review the strategic engineering of biophysical and biochemical cues in current scaffold designs that aid in restoring function to these preclinical VML injuries. We also discuss the successes and limitations of the three main biomaterial-based strategies to treat VML injuries: acellular scaffolds, cell-delivery scaffolds, and in vitro tissue engineered constructs. Finally, we examine several innovative approaches to enhancing the design of the next generation of engineered scaffolds to improve the functional regeneration of skeletal muscle following VML injuries.

## 1. Clinical Need: Volumetric Muscle Loss

A total of 65.8 million Americans suffer from musculoskeletal injuries annually, with treatment costs exceeding 176 billion dollars [1,2,3,4,5]. Although these injuries are not commonly life threatening, they profoundly impact the quality of life of patients. Musculoskeletal conditions are highly debilitating, comprising the second highest global volume of years lived with disability [6]. It is estimated that these injuries result in an additional 326 billion dollars annually in lost productivity [7]. 

Severe musculoskeletal injuries can lead to volumetric muscle loss (VML), where extensive musculoskeletal damage and tissue loss result in permanent loss of function [8,9]. VML injuries can result from sports injuries, surgical resection, and traumatic events such as car accidents and combat injury. In particular, musculoskeletal injuries sustained in combat present a unique challenge because they lead to the highest number of disabled war fighters and have the largest disability costs [10]. While the rate of combat mortality for U.S. Warfighters has dropped significantly since World War II, there has been a marked increase in the number of soldiers who suffer from extraordinary injuries, such as blast injuries, which impart extensive damage to the head, neck and extremities [11]. A total of 54% of all soldiers wounded on the battlefield suffer from at least one musculoskeletal extremity injury, with 53% of these injuries involving soft tissue damage [8,12]. Combat-related extremity injuries cause the greatest number of disabled soldiers [10]. Injured soldiers incur an average of 4.2 wounds, making extremity injuries the primary cause for hospitalization and evacuation from theater [10]. VML injuries also result in significant long-term disability that does not improve over time [13,14]. These extremity wounds also represent the largest projected disability costs of combat injuries [10,15]. The projected lifetime disability costs of a soldier with VML is $341,200 per individual [14]. Extremity injuries account for 69% of resource utilization, making them not only the most common injuries but also some of the most expensive to treat [15].

Due to the complex and large-scale nature of VML injuries, current treatment options remain limited and have substantial disadvantages. In the case of small-scale injuries or strains, muscle is capable of endogenous regeneration and complete functional restoration. However, this ability is abated in VML, where the native biophysical and biochemical signaling cues are no longer present to facilitate regeneration. These injuries are concomitant with denervation and the destruction of native vasculature, further limiting regeneration. Currently physical therapy is the only targeted treatment for VML injuries, and it has shown limited success in improving muscle strength [16,17,18]. The current standard of care for VML is autologous tissue transfer, where a muscle flap is excised from an undamaged muscle and grafted into the injury site [19,20,21,22]. This procedure is commonly referred to as a free functional muscle transfer (FFMT). While FFMT has been moderately successful in salvaging limbs and restoring some muscle function, muscle flaps remain unable to completely restore muscle function [22,23,24,25]. This procedure is also complicated and time consuming to perform and requires the expertise of skilled orthopedic and microvascular surgeons, which may limit its widespread use [19,26]. Additionally, a high instance of muscle flap procedures result in complications such as infection, graft failure, and donor site morbidity due to tissue necrosis [21,22,27,28]. Often a revisionary surgery or amputation of the affected limb is required [21,22,27,28]. Thus, a clinical need exists for the development of an alternative treatment that will restore function in VML injuries.

## 2. Skeletal Muscle Anatomy

**Figure 1:**
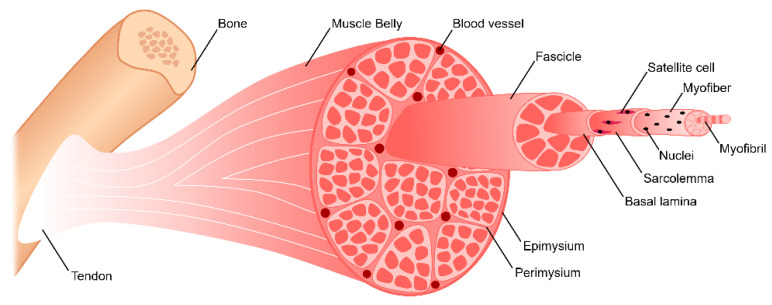
Skeletal muscle anatomy. Skeletal muscle is a highly aligned tissue with a hierarchically organized, cable-like structure.

Skeletal muscle is the most abundant tissue in the human body, making up approximately 40–45% of total body mass [29]. This tissue is primarily responsible for generating a series of discrete uniaxial forces that enable locomotion. It consists of hierarchically organized myofibers, vasculature, nerves, and connective tissue (Figure 1). Myofibers are elongated, cylindrical, multi-nucleated fibers that act as the functional unit of skeletal muscle. Myofibers are generated by the fusion of myoblasts to form multi-nucleated tubes, ranging in diameter from 10–100 μm depending on muscle location and function [29,30,31]. As these myofibers mature, their nuclei become oriented along the periphery just below the sarcolemma, the plasma membrane of the myofiber. Myofibers consist of myofibrils with repeating sarcomeres, the contractile unit of skeletal muscle. Sarcomeres contain contractile proteins thin filament actin and thick filament myosin. Within skeletal muscle tissue, parallel myofibers are bundled together to form fascicles, which are encased by perimysium. Subsequently, parallel bundles of fascicles are bundled together to form the muscle belly, which is surrounded by epimysium. Surrounding each myofiber is endomysial connective tissue known as the basement membrane and basal lamina. The perimysium, epimysium, and endomysium together provide structural support to the tissue while aiding in force transmission and synchronous contraction. The basal lamina is composed of proteins including type IV collagen, fibronectin, and laminin-2 [32,33]. It also consists of glycosaminoglycans (GAGs) and proteoglycans, such as heparan sulfate, which act as reservoirs for growth factors essential for myogenesis, including hepatocyte growth factor (HGF) and fibroblast growth factor 2 (FGF2) [32,33,34]. Heparan sulfate is also involved in HGF binding to its cell surface receptor, c-Met, by controlling the binding of HGF and regulating the cell’s mitogenic activity [35]. It also significantly enhances FGF2 signaling, binding to both the growth factor and its receptor, forming a ternary complex [36,37]. Structural evaluation of skeletal muscle basement membrane shows an aligned organization of architecture, including perimysial collagen bundles approximately 0.5–1 µm in diameter that run parallel to muscle fibers [33].

Just below the basal lamina and above the sarcolemma is where satellite cells (SCs), muscle-specific resident stem cells, are located [38]. In healthy skeletal muscle, SCs typically account for only about 2–7% of the total myonuclei [39]. They are identified by the expression of transcription factor paired box 7 (Pax7) and have been found to be necessary for skeletal muscle regeneration [40,41,42,43]. Upon injury, SCs leave their quiescent state and become activated to enter the cell cycle [44]. They proliferate and differentiate to form multi-nucleated myotubes, which mature to form myofibers. SCs are also capable self-renewing by maintaining a stem-like population [45]. A more detailed explanation of the role of SCs in skeletal muscle regeneration will be explored in Section 3. 

**Figure 2:**
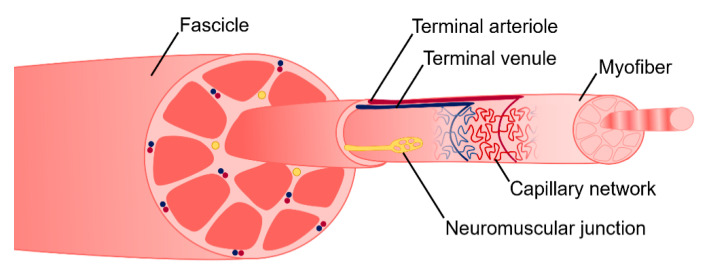
Anatomy of skeletal muscle vasculature and neuromuscular junctions. Arterioles, venules, and neurons run adjacent and parallel to myofibers.

To allow for voluntary locomotion, skeletal muscle is highly innervated. Motor neurons extend from the central nervous system and branch extensively throughout the muscle tissue to contact individual myofibers at a neuromuscular junction (NMJ) (Figure 2). The NMJ is the site at which an action potential from the motor neuron is converted to a muscle contraction. Contraction is initiated by acetylcholine release from the presynaptic axon, which subsequently binds to the myofiber and depolarizes the membrane. Membrane depolarization results in an action potential which travels down the length of the myofiber and initiates the release of calcium ions. Calcium binding within the myofibril results in an actin/myosin-mediated power stroke and muscle contraction. To meet its high metabolic demands, skeletal muscle tissue is also highly vascularized. An organized branching structure with capillary networks running parallel to the myofibers allow for optimal nutrient and oxygen exchange (Figure 2). Capillary networks in skeletal muscle are dense, with approximately 600 capillaries/mm [2,46]. This results in 40 µm distance between capillaries, and thus a 20 µm distance for oxygen diffusion [46].

## 3. Skeletal Muscle Regeneration

**Figure 3:**
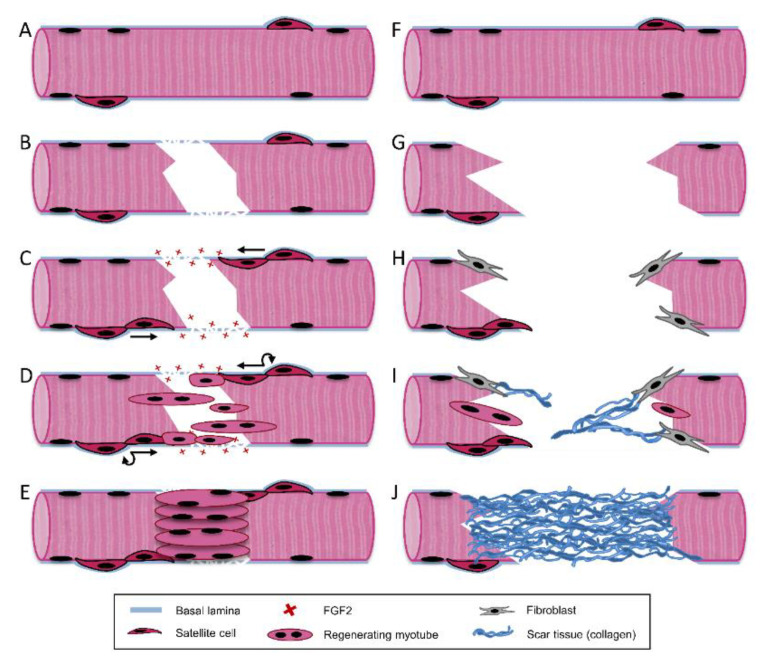
Skeletal muscle regeneration. Differences in regeneration following small scale skeletal muscle injuries (**A**–**E**) vs. volumetric muscle loss (**F**–**J**). Healthy muscle tissue (**A**) incurs a small-scale injury, which damages the myofiber and its surrounding basal lamina (**B**). The disrupted basal lamina releases sequestered growth factors including fibroblast growth factor 2 (FGF2) and satellite cells are activated, proliferating and migrating into the wound site along the basal lamina (**C**). Satellite cells begin fusing to form myotubes while simultaneously self-renewing (**D**). Resulting tissue is fully recovered, with aligned myotubes (**E**). Healthy muscle tissue (**F**) incurs a large-scale VML injury, which destroys the majority of native basal lamina and satellite cells (**G**). Without these cues, satellite cell-mediated regeneration is diminished, and fibroblasts begin infiltrating the wound (**H**). The injury is characterized by sparse and misaligned myoblast infiltration and collagen deposition (**I**), resulting in scar tissue formation and decrease in muscle function (**J**).

After acute injury, endogenous repair of skeletal muscle follows a highly coordinated regenerative process involving three separate but overlapping phases: destruction/inflammatory, repair, and remodeling (Figure 3A–E). 

### 3.1. Destruction/Inflammatory Phase

During the inflammatory phase, remodeling of damaged tissue and release of cytokines to promote regeneration are largely facilitated by immune cells including macrophages and neutrophils, and occurs within the first 1–2 weeks after injury [47]. Immediately upon injury damaged myofibers, blood vessels, and neurons undergo necrosis. This is due to membrane damage which permits an influx of extracellular calcium and triggers autodigestion via calcium-dependent proteases such as calpains [30,48,49,50]. In addition to cellular damage, disruption of the blood vessels and basement membrane surrounding myofibers also occurs upon injury. Mechanical injury to the basement membrane releases growth factors sequestered by proteoglycans within this ECM including HGF, FGF2, and transforming growth factor β (TGF-β) over a period of two weeks post-injury. (Figure 3C) [32,34,51]. The release of both intracellular contents and sequestered ECM growth factors, as well as activation of the complement cascade, stimulate resident mononuclear cells within the muscle tissue [30,32,50]. These cells then chemotactically recruit circulating leucocytes to the site of injury [52]. Neutrophils are the first sub-population of leucocytes to arrive, typically within the first few hours after injury [53]. These phagocytes are most active during the first 24 hours post-injury and aid in clearing necrotic cellular debris. While they act as a source of pro-inflammatory cytokines [54,55], they may also generate oxygen free radicals and ultimately induce tissue damage [56]. During the inflammatory phase TGF-β is released into the wound site by platelets. TGF-β acts as an immunomodulator, attracting and activating monocytes and macrophages to the injury [57]. Next, macrophages infiltrate and become the predominant cell type at the injury within several days. They can be classified into two distinct and sequential subpopulations: classically activated pro-inflammatory (M1) and alternatively activated anti-inflammatory (M2) macrophage [58,59]. Pro-inflammatory M1 macrophages are first to arrive after neutrophils and continue to phagocytose debris and release reactive oxygen species and pro-inflammatory cytokines [60,61]. These paracrine factors stimulate resident SC proliferation and mobilization [62,63]. M1 macrophages are eventually replaced by M2 macrophages, which have been associated with promoting SC exit from the cell cycle and commitment to differentiation [62,63,64]. Some of the secreted growth factors and cytokines that play an important role in this phase of regeneration are tumor necrosis factor-α (TNF- α), fibroblast growth factor (FGF), insulin-like growth factor (IGF), interleukin -1β (IL-1β), and interleukin 6 (IL-6) [54,55,60,61].

### 3.2. Repair Phase

The repair phase of skeletal muscle takes place 1–4 weeks post-injury and involves the activation and proliferation of SCs (Figure 3D) and their subsequent differentiation into mature muscle tissue (Figure 3E) [65]. Upon initial injury, disruption of the basement membrane initiates the release of heparan sulfate proteoglycan-bound growth factors within this matrix, including HGF and FGF2 [32,34,55,66,67]. HGF is released from the basement membrane by physical disruption and nitric oxide (NO)-mediated activation of matrix metalloproteinases (MMPs), which release HGF from the basement membrane [68,69,70,71]. HGF is released immediately upon injury, and its presence in muscle wounds peaks 2–4 days post-injury [51]. HGF has been shown to be the predominant growth factor capable of activating SCs to re-enter the cell cycle [35,68,72,73,74,75]. SCs express HGF’s receptor c-Met, allowing it to bind and stimulate SC activation [68,69,70,71,72]. In addition to promoting activation, HGF is also responsible for promoting SC proliferation and migration [55]. FGF2 is another growth factor responsible for the proliferation and migration of activated SCs [30,76,77,78]. FGF2 has been shown to be present in muscle wound fluid 2–8 days after injury, and peaks around 6–8 days [51]. In addition to HGF and FGF2, numerous other growth factors are responsible for promoting SC proliferation and migration, including TGF-β and platelet-derived growth factor (PDGF) [54,55]. TGF-β presence in muscle injuries has been shown to peak around 12–14 days post-injury [51]. SCs gives rise to quiescent SCs and committed myogenic progenitors, allowing for both self-renewal and the sub-population primarily responsible for muscle regeneration. SCs reside below the basement membrane on the periphery of skeletal muscle myofibers, which provides instructive biophysical contact guidance cues for their migration into the wound margin in response to injury [79,80]. SCs are guided by the basement membrane surrounding necrotic myofibers to facilitate aligned cell division, migration, and fusion into myofibers [80,81]. These mechanisms are driven in part by the aligned topographical cues provided by the basement membrane.

Next, muscle progenitor cells begin to differentiate, expressing myosin heavy chains (MyHCs) and fusing together to form multinucleated myotubes. Regenerating myofibers can be identified by their characteristic centrally located nuclei. While HGF, FGF2, TGF-β, and PDGF are essential during early phases of muscle regeneration, they are known to inhibit subsequent differentiation into mature muscle [73,82,83,84,85]. This highlights the importance of the highly regulated and temporal expression of growth factors throughout skeletal muscle regeneration. Other growth factors, including insulin-like growth factor 1 (IGF-1) and 2 (IGF-2), stimulate both proliferation and differentiation of SCs [54,55,86,87,88]. 

The success of muscle regeneration is also highly dependent on effective revascularization and reinnervation of the tissue, which occur concurrently during the repair phase. While SC-mediated activation, proliferation, and differentiation can take place in denervated muscle tissue, subsequent tissue maturation of newly regenerating myofibers is dependent on the presence of nerves [89]. Nerve activity has been shown to be crucial for skeletal muscle maturation because it provides electrical stimulation to the tissue [90]. Innervation also promotes the switch from fast to slow MyHC in regenerating muscle [90]. Additionally, revascularization of muscle tissue is paramount for successful regeneration. Upon injury, endothelial cells (ECs) sprout and form tubular structures in the direction of growth factor stimuli. Pericytes and smooth muscle cells are responsible for forming a layer over the ECs to stabilize the new vessels. Newly formed capillaries provide the oxygen required for aerobic metabolism, which is necessary for myofiber generation and maturation [30]. Additionally, the proximity of vasculature to SCs in vivo allows ECs to act upon SCs via paracrine growth factor signaling, stimulating their proliferation [91]. Growth factors play a critical role in promoting revascularization and innervation of skeletal muscle. For example, FGF2 has been shown to stimulate endothelial migration and sprouting, as well as pericyte and smooth muscle cell migration. FGF2 also stimulate the formation of more mature vessels than other proangiogenic GFs such as vascular endothelial growth factor (VEGF) [92]. Additionally, FGF2 has been shown to have neurotrophic activity, stimulating the synthesis and secretion of nerve growth factor (NGF) and promoting neuronal survival and outgrowth [54,93,94,95,96]. 

### 3.3. Remodeling Phase

The final phase of regeneration is the remodeling phase, which occurs 2–6 weeks post-injury [47]. This phase consists of regenerating myofiber maturation and ECM remodeling. The basement membrane acts to guide maturing myofibers [97]. Regenerating myotubes within the basal lamina may not fuse, causing the formation of small fiber clusters. Alternatively, fibers may fuse at only one extremity, causing forked fibers. Myofiber maturation is also highly dependent on revascularization of the tissue and the generation of neuromuscular junctions. Muscle begins to regain its contractile function during this phase. 

The formation and remodeling of scar tissue due to fibrosis also takes place during tissue remodeling. In addition to chemotactically recruiting inflammatory cells, TGF-β stimulates the synthesis of ECM molecules including fibronectin, collagens, and proteoglycans [57,98,99]. Fibroblasts are attracted to the wound site by TGF-β and increase the synthesis of ECM proteins [99]. TGF-β is also responsible for inhibiting ECM protease production and stimulating protease inhibitor production, making it responsible for the reconstruction of the basement membrane surrounding damaged myofibers [100]. Additionally, FGF2 is also known to stimulate fibroblast migration and proliferation [101]. Repair and replacement of connective tissue ECM that was damaged during injury is important because it provides stability for the regenerating muscle tissue. ECM is also important for enhancing muscle tensile strength as well as myofiber-tendon junctions [102]. However, an over-production of ECM often remodels into scar tissue and can inhibit muscle regeneration. The degree of scar tissue formation often increases with increasing severity and size of injury and is associated with poor functional outcomes. The role of scar tissue formation on tissue regeneration will be discussed further in Section 3.4 below.

### 3.4. Limited Capacity for Regeneration in VML Injuries

Although SC-mediated regeneration is effective in most muscle injuries, this is not the case in large-scale VML injuries. Due to the magnitude of these injuries, the basement membrane is typically compromised or destroyed, ablating native biophysical and biochemical cues necessary for SCs to facilitate regeneration (Figure 3G). With the basement membrane removed, the population of resident SCs is severely depleted. Thus, VML injuries appear to have a limited invasion of myoblasts into the injury site [30]. Additionally, the growth factor population that is sequestered within the basement membrane is also destroyed, which significantly limits their ability to guide regenerative processes such as SC-mediated myogenesis, the inflammatory response, revascularization, and reinnervation [103,104]. The biophysical cues provided by the basement membrane are also significantly limited upon VML injury. During normal regeneration, the basement membrane provides instructive biophysical contact guidance cues for SC aligned cell division and migration into the wound margin in response to injury [79,80,81]. However, when this ECM is removed in VML injuries, SCs have a limited capacity to migrate into the wound and undergo aligned cell division and myotube fusion. Lateral migration of SCs outside the basement membrane is more likely to occur in VML injuries, where the basement membrane has been disrupted [105]. In addition to limited contact guidance cues and signaling, these large-scale injuries also have a lack of mechanical support [106]. 

With limited muscle regeneration, fibroblast-mediated collagen I deposition dominates the healing response and generates non-functional scar tissue in the wound (Figure 3H–J). When M1 and M2 macrophage populations are depleted within a muscle injury, regeneration is impaired and fibrotic scar tissue is deposited [103]. Alternatively, a chronic inflammatory response can lead to dysregulation of growth factor expression and result in limited regeneration and fibrosis [104]. Scar tissue fills the void, bridging the remaining muscle fibers at each end of the injury to facilitate force transduction along the muscle [107]. In small-scale injuries, scar tissue can act as a conduit to aid in myogenesis. However, in VML injuries ECM deposition occurs quicker than myofiber formation and generates a dense scar tissue cap that inhibits myofibers from bridging the wound [29,107]. This can yield the formation of myotendinous junctions between adjacent myofibers and scar tissue [107]. Ultimately, fibrous tethering within VML injuries restricts torque production and range of motion, resulting in permanent loss of function in these injuries [16]. 

The extent to which VML injuries revascularize and reinnervate is vital for muscle regeneration and is also highly dependent on the extent of the injury. Often extensive skeletal muscle injuries include injury to the vasculature and neural networks surrounding the muscle tissue. Among the military population with VML lower limb injuries, 14% also had a nerve injury, and 5% had a vascular injury [14]. A nerve injury in conjunction with VML has also been observed in a murine animal model of skeletal muscle injury [108]. When a VML defect comprising 20% of the tibialis anterior (TA) muscle of Lewis rats was created, it yielded axotomy of 69% of the motoneurons innervating that muscle [108]. Without reinnervation, injured muscle tissues lack action potential-mediated muscle contractions, inducing atrophy. Reduced NMJ formation has also been associated with a depleted number of SCs in the injury [109]. Revascularization is also vital to VML injury, but often poses challenges. The degree to which skeletal muscle injuries revascularize depends on the severity of the injury; in larger VML defects there is limited revascularization because of a greater degree of scar tissue deposition [110]. Dense scar tissue can impede the ingrowth of neurons and vasculature and limit oxygen diffusion, yielding denervated and ischemic muscle [111,112]. 

## 4. Biomaterial Strategies for Skeletal Muscle Regeneration

To overcome the limitations of current clinical treatments for VML injuries, tissue engineered biomaterial scaffolds are under development with the goal of preventing scar tissue formation and enhancing functional muscle regeneration. Skeletal muscle tissue engineering and regenerative medicine present a promising therapeutic treatment by repairing or replacing the damaged muscle with a combination of instructive biomaterial scaffolds, biologically-active molecules, and cells [113,114]. Tissue engineered scaffolds are three-dimensional (3D) constructs that recapitulate the native ECM milieu, creating a synthetic microenvironment to locally control cellular functions and guide regeneration. To accomplish this, scaffolds must incorporate biophysical and biochemical cues that mimic native tissue composition, architecture, mechanics, and bioactive signaling. Biophysical cues include scaffold topography, porosity, and mechanics, while biochemical cues comprise the spatial and temporal control over the presentation of bioactive molecules. Scaffolds are made of synthetic or natural materials with demonstrated biocompatibility such that the scaffold will not cause toxicity, injury, or immunological rejection when implanted in living tissue. Biocompatible scaffolds allow for the incorporation of cells and biologically-active molecules, such as proteins, peptides, growth factors, cytokines, transgenes, and messenger ribonucleic acid (mRNA). 

**Figure 4:**
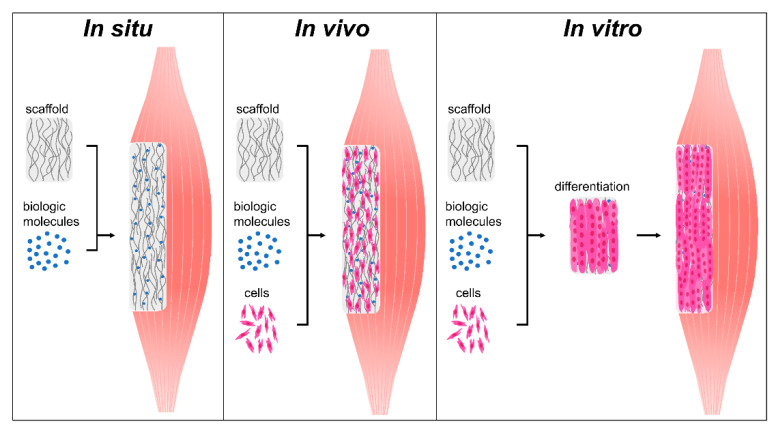
Skeletal muscle tissue engineering approaches. In situ tissue engineering relies on endogenous regeneration guided by an acellular scaffold with instructive biophysical and biochemical cues. In vivo tissue engineering transplants a scaffold, biologic factors, and cells, creating a synthetic niche and delivering cells to aid in regeneration. In vitro tissue engineering utilizes the same factors but first differentiates the cells in vitro into a functional construct prior to implantation.

Skeletal muscle tissue engineering can be broadly classified into one of three approaches: in situ, in vivo, and in vitro tissue engineering (Figure 4, Table 1) [29,115]. These treatments range in complexity and can act by enhancing endogenous regeneration or by generating engineered tissues to replace damaged muscle. In situ tissue engineering involves the implantation of an acellular biomaterial scaffold into the injury that can direct endogenous regeneration. Strategic engineering of biophysical and biochemical cues allows the scaffold to instruct host cell recruitment, activation, proliferation, and differentiation. Slightly more complex, in vivo tissue engineering involves seeding instructive biomaterial scaffolds with cells immediately prior to transplantation, where they can then participate in regeneration. While this approach limits the manipulation of cells prior to transplantation and preserves their efficacy, it can leave them susceptible to low viability, retention, and immune rejection [29,116,117]. Finally, in vitro tissue engineering involves the development and implantation of a functional tissue engineered construct. This is achieved by combining scaffolds, biological factors, and cells and culturing these constructs in vitro until the cells differentiate into contractile myofibers. Differentiation is often achieved through a combination of biochemical cues, mechanical stimulation, and electrical stimulation. While in vitro tissue engineered constructs have greater functionality prior to implantation than those developed through in situ and in vivo techniques, they have several significant drawbacks. While they display some functionality, these contractile forces are often significantly lower than what is seen in native muscle tissue [106]. Additionally, due to oxygen and nutrient diffusion limitations these constructs are often size-limited or require the development of a complex vascular network to support extended cell viability. Herein we review past and current skeletal muscle tissue engineering strategies, with a focus on the use of instructive biomaterial scaffolds. This review will not cover scaffold-free approaches to treating VML, such as rehabilitation regimes, autologous grafts, or minced muscle grafts. It will also not include cellular, drug, gene, or growth factor injections unless they are delivered using a biomaterial carrier. Additionally, this review will focus exclusively on the treatment of muscle injuries and not include treatments for genetic diseases such as Duchenne muscular dystrophy. While this review focuses on VML resection injuries, it also evaluates muscle injuries induced from critical limb ischemia (CLI), crush, and myotoxin injuries, which present a different pathophysiology and capacity for functional recovery than VML injuries [118]. Preclinical and clinical in situ, in vivo, and tissue engineering strategies will be reviewed, with a focus on the biophysical and biochemical cues of these scaffolds that guide regeneration.

### 4.1. In Situ Strategies: Acellular Scaffolds to Promote Endogenous Regeneration

In situ tissue engineering utilizes acellular biomaterial scaffolds to direct endogenous regeneration. Tissue engineered scaffolds employ biophysical and biochemical cues to recapitulate native ECM structure, creating a synthetic microenvironment to locally control host cellular functions and guide regeneration. Implantation of an acellular scaffold offers unique advantages over cell-based strategies, including faster and simpler fabrication and storage. By not delivering cells the culture time for these scaffolds is eliminated, yielding faster fabrication, streamlined delivery workflows and operations, the potential for long term storage and off-the-shelf capabilities. Additionally, these factors result in the production of scaffolds that often have lower regulatory barriers and a quicker path to commercialization (Table 1) [120]. For this strategy to be effective, strategic engineering of the biomaterial scaffold is paramount to develop a synthetic niche capable of directing endogenous regeneration. This section will focus on the strategies utilized for skeletal muscle scaffold development, with a focus on biomaterial selection and biophysical and biochemical cues used to modulate these materials.

#### 4.1.1. Biomaterial Selection: Synthetic, Natural, and Hybrid Polymers

Most biomaterials fall into one of two classes, synthetic or natural, which both offer distinct advantages and drawbacks. Synthetic scaffolds are easy, consistent, and inexpensive to fabricate and can be manufactured to have detailed geometries down to the nanoscale. They can also be engineered to have precise and tunable degradation profiles and mechanical properties. Conjugation of biomolecules such as growth factors is also possible, and their release from the scaffolds can be finely tuned by altering conjugation strategies or scaffold degradation rates. Synthetic polymers offer additional benefits, such as the ability to be electrically conductive [121,122,123]. Synthetic scaffolds that have been used for skeletal muscle tissue engineering include poly(ε-caprolactone) (PCL), poly(glycolic acid) (PGA), polylactic acid (PLA, PLLA), and copolymer poly-lactic-*co*-glycolic acid (PLG, PLGA), polyurethane (PU), polyethylene glycol (PEG), and polypropylene (PP). However, use of synthetic biomaterials comes at a cost. These polymers are often associated with low cell attachment, limiting their use without functionalization of a natural biopolymer. Additionally, synthetic scaffolds have limited biocompatibility and have been shown to elicit a pro-inflammatory immune response upon implantation [116]. 

In contrast, natural polymers are highly biocompatible and contain native signaling cues which aid in promoting cellular attachment, proliferation, and differentiation. They also contain native functional groups suitable for growth factor conjugation and are naturally degraded upon implantation. Natural biopolymers include collagen, fibrin, alginate, laminin, silk fibroin, hyaluronic acid (HA), decellularized ECM, chitosan, keratin, and gelatin. While biopolymer scaffold porosity, topography, and mechanics can be modified, there is less precision and tunability than with synthetic scaffolds. Biopolymers are also subject to inherent biologic variability due to material sourcing. Both synthetic and natural biomaterials can be made into an ideal scaffold through strategic incorporation of biophysical and biochemical cues designed to create a synthetic microenvironment conducive to skeletal muscle tissue regeneration. 

#### 4.1.2. Biophysical Cues

**Figure 5:**
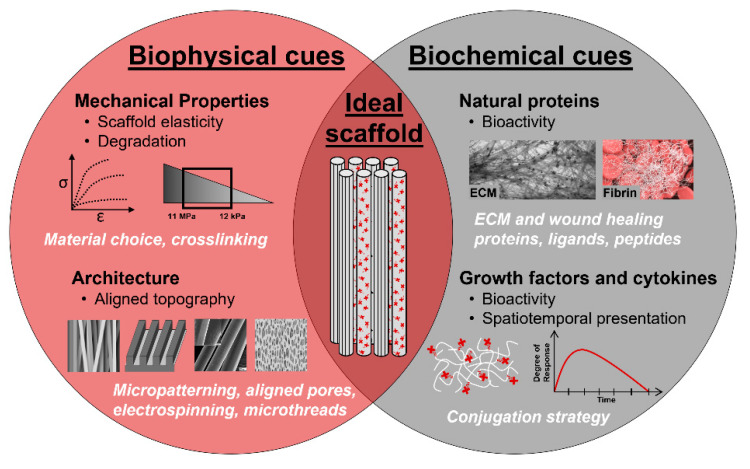
The role of biophysical and biochemical cues in designing biomaterials for skeletal muscle tissue engineering. Biophysical cues include scaffold mechanics, degradation and architectural morphology. Biochemical cues include exploiting natural molecules, and spatiotemporal delivery of biomolecules such as growth factors. In concert, biophysical and biochemical cues allow for the generation of scaffolds that effectively recapitulate a cellular microenvironment conducive for regeneration. Image concept adapted from [126].

An ideal biomaterial scaffold should match native tissue mechanical properties, degrade at a rate that matches the rate of new tissue regeneration, and contain 3D topographical features and porosity to direct cellular alignment and allow for cellular infiltration. These features can be accomplished through the incorporation of instructive biophysical cues (Figure 5). Scaffold strength and stiffness should be optimized to match native tissue mechanics to create a synthetic niche that exposes cells to physiologically relevant forces. Myoblasts respond to mechanical stimuli through mechanotranduction, informing their proliferation, adhesion, and differentiation. Substrate stiffness does not affect the propensity for myoblasts to assemble into myotubes, but it was shown to have an important role on the development of myosin/actin striations [124]. Myoblasts cultured on substrates with a modulus of 12 kPa, which matches the elasticity of native resting muscle tissue, were found to have significantly increased striations, indicating a more functional and mature cellular phenotype [124]. Scaffold biodegradation is another biophysical cue that must match the kinetics of skeletal muscle regeneration and new tissue ingrowth [125]. Rapid degradation can lead to voids within the tissue and compromised regeneration, while slow degradation can invoke a chronic inflammatory response, scar tissue deposition, and encapsulation [126]. Both scaffold mechanics and degradation are commonly controlled by material choice and crosslinking. Hydrogels, sponges, fibers, composites and devitalized ECM are among the most commonly exploited conformations for biomaterial scaffolds. These systems are all able to create a 3D environment that provides suitable porosity, topographical cues, and mechanical properties to support tissue regeneration.

##### Decellularized ECM

One of the most commonly exploited acellular scaffolds is decellularized ECM. Decellularized ECM is a scaffold prepared by removing cells from source mammalian tissue or whole organs, leaving behind the native ECM with preserved structural and chemical composition. Decellularized ECM has been used as a biomaterial because it contains the native ligands, ECM proteins, and growth factors found in skeletal muscle which are known to play an instrumental role in SC chemotaxis and proliferation, the inflammatory response, myotube differentiation and, ultimately, functional tissue regeneration [63,127,128,129]. These ECM molecules include collagens, laminin, fibronectin, heparin sulfate, chondroitin sulfate, HA, VEGF, FGF2, and TGF-β [130,131,132,133]. The goal for these scaffolds is that upon implantation into a muscle injury, they will become infiltrated by immune cells that degrade the scaffold, releasing native growth factors and ECM proteins that promote host cell infiltration [63]. Decellularized ECM is harvested from a variety of source tissues, including dermis, skeletal muscle, small intestinal submucosa (SIS), and urinary bladder matrix (UBM) [127,128]. Different source tissues provide varying structural organization and chemical composition [134]. The process by and extent to which ECM is decellularized can also yield varying physical and chemical properties, and contribute to the varying degrees of remodeling seen after implantation [135,136,137]. This may explain the conflicting reports in the literature of decellularized ECM inflammatory response, induction of fibrosis, and ability to promote muscle fiber regeneration upon implantation.

Early studies investigating the use of xenogeneic SIS and homologous muscle ECM found that implanted scaffolds promoted a strong angiogenic response despite variable contractile function and the persistent deposition of collagenous connective tissue [138,139,140]. In one study, Badylak’s group observed that upon implantation into rodent partial resection models, both SIS and muscle ECM scaffolds induced constructive remodeling characterized by robust mononuclear cell infiltration and myogenesis, although no contractile analysis was performed to evaluate muscle function [134,141]. Corona et al. implanted a syngeneic muscle ECM scaffold into a partially resected rat TA muscle and saw recovery of one third of the original force deficit was restored after two months post-injury, despite histological analysis showing overwhelming fibrosis at the implantation site [142]. The authors hypothesize that the ECM scaffold prevented muscle fiber damage and acted as a structural reinforcement to transmit forces across the injury. Another study evaluated a syngeneic muscle ECM scaffold to treat a rodent TA VML defect model and found that at two weeks post-implantation, the scaffold elicited a pro-inflammatory response with a large quantity of macrophages surrounding the implant [143]. At eight weeks after treatment little to no myosin^+^ muscle fibers were present, and increased collagen 1 was observed. Upon stimulation, TA muscles treated with muscle ECM demonstrated a 17% increase in torque compared to those with an injury and no treatment. More recent studies utilizing a UBM ECM scaffold to treat rodent and porcine VML injuries found that injuries treated with scaffolds demonstrated limited myogenesis, fibrotic deposition, and chronic functional deficits at terminal timepoints [144,145]. Discrepancies in the existing literature regarding whether or not ECM scaffolds promote constructive remodeling in preclinical models may be due in part to differences in ECM sources, decellularization protocols, differences in anatomy, and the severity of preclinical VML models.

Decellularized ECM is the first tissue engineered scaffold to be clinically evaluated for treating VML injury. A 2010 case study treated a persistent combat-induced quadriceps injury 3.5 years post-injury with an acellular porcine SIS ECM scaffold and subsequent physical therapy [17]. At 16 weeks post-operatively a modest improvement in isokinetic muscle function was demonstrated, as well as new soft tissue observed via computed tomography (CT) at 36 weeks post-operatively. A 2014 study by Sicari et al. evaluated the use of UBM ECM in five male patients with extremity VML injuries incurred at least six months prior that maintained a minimum of 25% functional deficit compared to the contralateral uninjured limb [120]. At 6 months post-surgery magnetic resonance imaging (MRI) and histological evaluation of biopsies both showed the presence of vascularization and islands of muscle cells. Furthermore, three of the five patients showed a 20% functional improvement of the affected limb. A follow-up study of eight patients with VML, including the five from the previous study [120], used electrodiagnostic evaluations with nerve conduction studies (NCS) and needle electromyography (EMG) to demonstrate restoration of nerve tissue as it relates to variable functional outcomes in ECM-treated VML injuries [146]. The study found that five of the eight patients treated with ECM scaffolds demonstrated improvements in electrical activity evaluated through NCS and EMG as well as improved muscle strength, compared to the pretreatment condition. Most recently, a 13-patient study was conducted to evaluate the ability of ECM bioscaffolds and physical therapy to improve force production, range-of-motion, and function in a range of VML extremity injuries [147], which also included five patients from the previous study [120]. Patients demonstrated an average improvement of 37.3% in strength 27.1% in range-of-motion tasks and 271.8% in functional task performance at six months post-operatively. The authors acknowledged that debridement of scar tissue during surgery and the effect of mechanical transduction via the ECM scaffold may have contributed to these modest functional gains. They also observed the formation of muscle tissue at the injury site through histology of biopsied tissue and MRI or CT imaging. Despite some promising results, the clinical use of decellularized ECM remains limited due to variable outcomes among patients and the limited understanding of the mechanisms by which these acellular scaffolds mediate muscle regeneration.

##### Hydrogels, Sponges, and Meshes

Hydrogels, sponges, and meshes are used as alternative acellular scaffolds to decellularized ECM because they allow for more precise control of the scaffold material, mechanics, degradation, and porosity. Porosity is a critical biophysical cue to control in tissue engineered scaffolds because of its role in permitting cellular infiltration and oxygen and nutrient diffusion. Porosity is commonly achieved through the use of hydrogels, sponges, and fibrous meshes. Pore sizes typically range from 10 to 500 µm, and larger macropores typically permit greater cell viability and migration [148]. Injectable in situ polymerizing collagen and decellularized ECM hydrogel scaffolds were evaluated for their ability to treat critical limb ischemia (CLI) in a rat hindlimb ischemia model [149]. They found that decellularized ECM hydrogels promoted increased the number of MyoD^+^ cells recruited to the injury and blood vessel density compared to the collagen hydrogel. More recent work delivering a laminin 111-enriched fibrin hydrogel to a murine VML defect demonstrated an infiltration of macrophages and ECs into the hydrogels at two weeks post-injury, but did not report increases in peak isometric torque at four weeks post-injury compared to the untreated negative control [150]. Sponge-based scaffolds can be generated by freeze-drying polymer solutions or hydrogels, creating a porous microstructure. Freeze-dried collagen sponges implanted into a partial resection of the vastus lateralis muscle in a rabbit model qualitatively demonstrated less scar tissue formation and a greater number, diameter, and length of myofibers at 24 weeks post-injury compared to the untreated control [151]. Lyophilized, highly porous sponges made of gelatin, collagen, and laminin 111 and crosslinked with 1-ethyl-3-(3-dimethyl aminopropyl) carbodiimide (EDC) have also been evaluated in a 10% resection of the gastrocnemius-soleus complex of mice [152]. At two weeks post-injury protein lysates from sponge-treated muscles showed significantly higher expression of MyoD and desmin compared to untreated muscles, suggesting an increase in myogenic activity at the injury site due to scaffold-mediated regeneration. In addition to hydrogels and sponges, fibrous meshes are also highly porous scaffolds that permit cellular infiltration. Fibrous meshes of PLLA with an average fiber diameter of 150 μm and pore size of 50–100 μm demonstrated host cell infiltration in rat TA VML defects. Histological evaluation of TA muscles at one, two, three, and four weeks demonstrated an influx of Von Willebrand factor (vWF)^+^ ECs and Pax7^+^ muscle progenitor cells into the fibrous mesh scaffolds over time [153]. While acellular hydrogels, sponges, and meshes permit cellular infiltration upon injury into VML defects, these scaffolds have not demonstrated aligned myofibers or significant gains in muscle function. This may be due in part to the lack of instructive topographical alignment cues that these scaffolds provide due to their isotropic nature. This has motivated a thrust of skeletal muscle tissue engineering research that focuses on the development of anisotropic scaffolds with aligned architectural features. 

##### Aligned Scaffolds

Incorporation of instructive biophysical cues such as anisotropic surface topography is a commonly exploited technique to promote cell alignment in skeletal muscle tissue engineering. Myoblast alignment is an essential step towards myotube formation, which is guided in vivo by ECM structure and micron-scale grooves between adjacent muscle fibers. Many strategies have been explored to create scaffolds with anisotropic surface topography, including patterned substrates [154,155,156,157,158,159,160,161]. electrospun fibers [122,123,155,162,163,164,165], microthreads [166,167,168], and aligned pores [169,170,171]. A review describes these methods in detail and their ability to align and differentiate myoblasts in vitro, as this falls outside of the scope of this review [172]. These methods are also commonly employed to generate in vitro tissue engineered skeletal muscle, which will be discussed in Section 4.3. Nakayama and colleagues evaluated the therapeutic benefit of aligned nanofibrillar collagen scaffolds and rehabilitative exercise on the treatment of VML [173]. Ablated murine TA muscles were treated with either aligned or randomly oriented collagen nanofibers, and animals were randomly assigned to either voluntary cage running or no rehabilitation regime during recovery. After 21 days post-treatment, muscle treated with both random and aligned nanofibers exhibited significantly higher myofiber cross sectional area than those left untreated or treated with a decellularized ECM scaffold. Additionally, they noted significantly higher perfused vascular density in muscles treated with the aligned nanofibers compared with those treated with the randomly-oriented nanofibers. This study warrants further investigation of anisotropically aligned scaffolds. Use of these scaffolds in conjunction with biochemical cues and cells will be discussed in later sections of this review.

#### 4.1.3. Biochemical Cues

Biochemical cues are often strategically incorporated into scaffolds for skeletal muscle tissue engineering as a method to further regulate cellular functions including survival, attachment, proliferation, migration, and differentiation into myotubes. These include biologically active molecules such as proteins, peptides, growth factors, cytokines, transgenes, and messenger ribonucleic acid (mRNA). Strategic design of biomaterial-based scaffolds must take place to protect these molecules from degradation, maintain their native conformation, and preserve their bioactivity. Biomaterial scaffolds should also be designed to carefully control spatiotemporal presentation, biomolecule release kinetics, and local concentrations. For example, genetic substances can be delivered via viral or non-viral vectors, such as liposomes and synthetic particles, engineered to translocate into the cell or nucleus. 

##### Growth Factors and Cytokines

Growth factors and cytokines are among some of the most commonly investigated biologic molecules to treat skeletal muscle injuries due to their instrumental role in facilitating native regeneration (reviewed in Section 3) [54]. Early clinical trials using bolus injections of growth factors such as VEGF and FGF2 to treat cardiovascular disease had limited success [174,175,176]. This is likely attributed to the bolus delivery method, which provided initial supraphysiological growth factor concentrations followed by rapid degradation, preventing sustained presentation of these factors for the necessary time frame [177,178]. Delivering growth factors via biomaterial carriers prevents their denaturation and mediates their release, which is controlled by scaffold degradation and/or diffusion through the matrix. They are often incorporated into biomaterials through physical entrapment, ionic interactions, or covalent coupling [126,178]. Engineering these scaffolds to control growth factor release kinetics allows for the delivery of optimized concentrations, localized delivery, and increased therapeutic efficiency. 

Several researchers have investigated the ability of growth factor-loaded scaffolds to promote skeletal muscle regeneration in ischemic and VML injury models [153,179,180,181,182,183,184,185,186,187,188,189,190]. VEGF-loaded hydrogel scaffolds have been investigated for their ability to promote angiogenesis in hindlimb injuries [179,180,181,182]. Silva et al. confirmed that VEGF delivered to a TA ischemic murine injury via alginate hydrogel was present at physiologically relevant levels for up to 15 days post-injection, compared to only three days after delivery of VEGF via bolus injection [179]. Sustained delivery of VEGF via alginate hydrogels resulted in significantly higher blood vessel density and tissue perfusion compared to no treatment and bolus VEGF delivery. Another study evaluated the sustained delivery of VEGF from alginate hydrogels for the treatment of an ischemic murine TA injury [180]. After seven days post-injury, VEGF delivery resulted in 50% innervated motor end plates compared to 5% in the blank alginate gel, likely due to the significant increase in glial-derived neurotrophic factor (GDNF) and NGF expression levels compared to uninjured muscle. Hydrogel-mediated VEGF also resulted in significantly higher number of vessels (CD31^+^) and mature vessels (smooth muscle actin^+^) after 14 days compared to blank hydrogels. To generate a more controlled release of VEGF from alginate hydrogels, Lee et al. encapsulated VEGF within PLGA microspheres, creating a sustained release of VEGF over three weeks [181]. These composite scaffolds generated significantly higher platelet endothelial cell adhesion molecules (PECAM) expression than bolus VEGF or VEGF-loaded alginate hydrogels, indicating the formation of functional microvessels. Another study evaluated the ability of VEGF-coated collagen matrices to stimulate repair in a rabbit lower leg osteotomy with soft tissue contusion injury, and found VEGF scaffolds resulted in 73% recovery of muscle strength compared to 53% recovery of no treatment control group after 30 days post-injury [182]. 

IGF-1 has also been used to treat VML injuries because of its important role in stimulating myoblast survival, proliferation, and differentiation [153,183]. Hammers et al. found that a controlled release of IGF-1 from (PEG)ylated fibrin gels implanted into a murine hindlimb ischemia injury stimulated significantly higher force production 14 days post-injury compared to bolus IGF-1 and blank hydrogel treatments [183]. Other researchers implanted IGF-1-loaded gelatin porous sponges into TA muscle of rats and after two weeks found a four-fold increase in the number of Pax7^+^ infiltrated cells and significantly greater number of muscle fibers compared to control blank hydrogels [153]. 

Another commonly utilized growth factor for promoting skeletal muscle regeneration is FGF2 [153,184,185,186]. In a rabbit hind limb ischemia injury model, FGF2-loaded gelatin hydrogels had significantly greater tissue blood flow, number of arterioles, and vascular density four weeks after treatment [184]. Other researchers have delivered sustained FGF2 release from ionic gelatin hydrogels covalently crosslinked with PLG, which yielded significantly greater capillary density (CD31^+^) and blood flow compared to bolus FGF2 injection at eight weeks after ischemic hindlimb injury [185]. Murine TA muscles implanted with FGF2-loaded gelatin sponges found a significant increase in the number of Pax7^+^ infiltrated cells and significantly greater number of muscle fibers compared to control blank hydrogels [153]. 

Our laboratory delivered HGF adsorbed to EDC-crosslinked fibrin microthread scaffolds in a murine VML defect [187]. Surgical resection of 30 mg of the TA muscle yielded approximately 50% reduction in force immediately after injury, and was immediately filled with fibrin microthreads that were EDC crosslinked and passively adsorbed with 40 ng/mL of HGF. At 60 days post-injury, this treatment resulted in an increase of over 200% of twitch and tetanic force production, compared to the 150% and 130% increases seen from treatment with fibrin microthreads or fibrin gel with no HGF delivery. HGF fibrin microthreads resulted in a significantly higher recovered force production than injuries that received no treatment or treatment with a fibrin hydrogel alone. This work has been recently acknowledged as the only study of growth factor-based repair that evaluated functional recovery in an appropriate VML injury model [191]. Histological analysis showed myofibers adjacent to implanted EDC crosslinked microthreads, indicating that the aligned microthread architecture likely guides myofiber ingrowth and alignment [187]. This motivates the future development of scaffolds with biophysical cues such as aligned topography in conjunction with the delivery of biochemical cues such as growth factors. Overall, the delivery of a single myogenic or angiogenic growth factor to skeletal muscle injuries yields improvements in regeneration, but it remains unclear which growth factors, concentrations, and delivery strategies yield the best results, and warrants further investigation.

Toward the goal of recapitulating in vivo regeneration, several studies also assessed the synergistic presentation of multiple growth factors. While delivery of a single growth factor has shown promising results for promoting skeletal muscle regeneration and angiogenesis, this strategy represents a drastically simplified version of the complex, spatiotemporal presentation of multiple factors during regeneration. Through the development of more complex biomaterials systems, the release kinetics of multiple factors in a spatiotemporal manner that mimics in vivo presentation and concentration will likely enhance regenerative outcomes [126,192]. The first preclinical work evaluating delivery of multiple growth factors for skeletal muscle repair investigated the co-stimulatory effect of IGF-1 and VEGF delivered to ischemic rodent hindlimbs via an injectable alginate hydrogel [188]. Despite the limited control over release kinetics that this scaffold provided, the co-delivery of 3 µg each of these factors from the alginate hydrogel stimulated significantly higher blood perfusion seven weeks after ligation compared to the blank hydrogel and bolus IGF-1/VEGF treatments. Injuries treated with the IGF-1/VEGF hydrogel also had significantly larger myofiber diameters and number of centrally located nuclei compared to blank gels, which are hallmarks of regenerating muscle. Another study evaluated the therapeutic potential of stromal cell-derived factor-1 α (SDF-1α) alone or in combination with IGF-1 to treat an ischemic skeletal muscle injury in rodents [189]. Co-delivery of these factors in a PEGylated fibrin hydrogel yielded significant improvements in revascularization (CD31^+^ cells/fiber) and functional recovery (maximum tetanic force production) at 14 days post-injury compared to treatment with blank hydrogel, which was not achieved by the delivery of SDF-1α alone. Matsui et al. investigated gelatin hydrogel granules to deliver FGF2 alongside a mix of growth factors isolated from platelet-activated platelet rich plasma (PRP), which included PDGF, VEGF, TGF- β, and FGF2 [190]. One week after implantation into a murine hindlimb ischemic injury the combination treatment yielded a significantly higher number of blood vessels compared to no treatment, which was not achieved by delivery of FGF2 or the PRP-isolated growth factor mixture alone. The combination treatment also significantly enhanced blood reperfusion compared to hydrogels with just FGF2 or PRP-isolated growth factors. As biomaterial scaffolds advance to allow for more precise spatiotemporal delivery of multiple growth factors, this strategy will better mimic the complex in vivo temporal presentation during regeneration and will likely yield greater therapeutic, functional outcomes.

##### Genetic Substances

Another thrust of research focuses on the delivery of genetic material including cDNA and mRNA. Gene therapy strategies transfer genetic material into host cells to treat genetic diseases or injury. This treatment often utilizes engineering of viral and non-viral vectors to safely and efficiently transfer genetic material into cell nuclei. These strategies include adenovirus, adeno-associated virus, retrovirus, lentivirus, liposomes, synthetic particles, and polymer-based scaffolds, and display varying degrees of immunogenicity and transfection efficiency. These approaches are typically limited to injections that do not control vector spatiotemporal presentation. To make gene therapy treatments more effective, researchers recently utilized biomaterial-based delivery systems to provide localized and sustained delivery of these therapies. Biomaterial scaffolds can help deliver genetic cargo by preserving genetic structures, protecting them from nuclease-mediated degradation and controlling their release from the scaffold. By modulating scaffold properties such as molecular weight, porosity, or crosslinking, a localized and sustained release of genetic material can be mediated through diffusion or scaffold degradation [193,194]. This delivery strategy has the potential to increase transfection efficiency and expression, ultimately improving the therapeutic effectiveness of these treatments.

One study evaluated the delivery of adenoviral vectors and plasmids encoding FGF2 and FGF6 transgenes delivered in collagen-gelatin scaffolds to excisional quadriceps defects in rats [195]. At 21 days after injury they found that treatment with FGF2 transgenes increased the arteriole density by 11-fold and myotube marker CD56 expression 20-fold compared to controls. They also note that the delivery of recombinant FGF2 protein was unable to produce equivalent responses, highlighting the benefit of a gene delivery strategy for treating skeletal muscle injury. Another gene therapy study delivered plasmid FGF4 cDNA within a gelatin hydrogel scaffold to treat hindlimb ischemia in rabbits [196]. The hydrogel preserved the plasmid structure allowing for improved transfection efficiency compared to naked FGF4 gene. Ischemic injuries treated with hydrogel-FGF4 had significantly less severe tissue damage and more pronounced vascular responsiveness to adenosine at four weeks compared to injuries treated with a naked FGF4 gene. 

An alternative strategy to the delivery of plasmids and viruses is mRNA-based delivery. This method is advantageous because it does not present the risks of genome integration or insertional mutagenesis that other gene therapy strategies have, but it can be limited based on the conventional delivery method of direct solution-based injection. Biomaterial-based delivery of mRNA allows for localized and controlled release of cargo. Recently, Zaitseva et al. evaluated the delivery of modified mRNA encoding HGF through crosslinked nanofibrillar collagen scaffolds to a murine skeletal muscle injury [197]. After 20% ablation of the TA muscle, scaffolds were implanted to fill the defect void. At two weeks after implantation, capillary density (CD31^+^) was observed in injuries treated with the HGF mRNA than by control scaffolds loaded with firefly luciferase-mRNA. As the use of biomaterial-based delivery of genetic substances continues to be implemented, it is likely that this strategy will continue to be investigated for skeletal muscle regeneration. However, cost, immunogenicity, and safety remain concerns regarding this relatively new approach [114].

##### Small Molecules

Small molecules are another class of biologic molecules that have been investigated for skeletal muscle regeneration. These organic compounds are typically under 800 Da in size which allows them to readily diffuse across the cell membrane, unlike macromolecules [198]. Small molecules can be produced using synthetic chemistry, allowing for diverse structures and lower manufacturing costs compared to larger molecules such as recombinant growth factors. They work by regulating biological targets such as receptors or enzymes to modulate specific cellular effects. These effects can be reversed and temporally controlled to allow for rapid activation or inhibition. However, small molecules are often delivered orally or via direct injection and the risk of off-target events where proteins with similar conformations are affected can be problematic [198]. To address this, biomaterial-based delivery of small molecules to localize treatment to target tissues is something that is recently being explored. One study investigated the use of a PLGA thin film to deliver FTY720 to a full thickness VML defect in the spinotrapezius muscle of mice [199]. FTY720 is a small molecule agonist of sphingosine-1-phosphate (S1P), a bioactive signaling lipid produced by red blood cells, platelets, and ECs [199]. Receptors of S1P are highly expressed on anti-inflammatory monocytes and have been shown to leverage pro-regenerative effects [200]. They demonstrated that, three days after injury, localized delivery of FTY720 had a higher percentage of anti-inflammatory monocyte and macrophage infiltration into the injury compared to defects treated with the PLGA film alone [199]. They also found significantly higher blood vessel density (CD31^+^) in the defect region in mice treated with FTY720 compared to those treated with a blank scaffold control. Another group also found angiogenic effects with the biomaterial-based delivery of small molecule CEP03, derived from CEP (ω-[2-carboxyethyl]pyrrole) protein adducts [201]. CEP protein adducts are a product of lipid oxidation and accumulate during inflammation and wound healing, but as-is face limitations of protein delivery including high cost and manufacturing challenges. CEP03 was encapsulated in Matrigel, an ECM generated by carcinoma cells in vitro, injected intramuscularly into the gastrocnemius muscle of mice with hind limb ischemia. Fourteen days after treatment with CEP03, a significant increase in relative mean perfusion ratio (ischemic/unoperated leg) and microvessel density (CD31^+^) was observed compared with injuries treated with Matrigel alone. Ongoing work developing biomaterials to spatiotemporally deliver small molecules has the potential to increase their therapeutic potential and make this a more commonly investigated approach to treating skeletal muscle injury.

### 4.2. In Vivo Strategies: Utilizing Biomaterials to Improve Cell Delivery

While acellular in situ tissue engineering strategies reviewed in Section 4.1 have demonstrated success in promoting endogenous skeletal muscle regeneration through recruitment of host cell populations, there are conflicting studies described in the literature that question whether this strategy will facilitate full functional recovery after the large-scale injuries incurred from VML [63,202]. Corona et al. hypothesized that restricted host SC infiltration into the VML defect is primary limitation to the success of this strategy, as they noted a low number of Pax7^+^ SCs within a defect treated with an acellular ECM scaffold [202]. Thus, one of the most commonly explored approaches for skeletal muscle regeneration involves the delivery of cells. While early research focused on the direct intramuscular injection of cell suspensions, this simplistic delivery technique has several significant limitations including poor retention, survival, and immune rejection of transplanted cells [29,203]. However, the recent development of biomaterial-based delivery systems with strategic biophysical and biochemical signaling cues creates a synthetic microenvironment conducive for cell survival and engraftment upon transplantation. The first study to evaluate the efficiency of SCs delivered via a polymer scaffold found 3-fold higher engraftment of cells into host muscle compared to those delivered via conventional direct injection [204]. Biomaterial delivery systems may also regulate cell fate, stemness and, ultimately, participation in regeneration. In vivo tissue engineering involves seeding instructive biomaterial scaffolds with cells immediately prior to transplantation, where they can then provide host cells with paracrine signaling and/or participate in regeneration [116]. By limiting the culture time and manipulation of cells prior to their transplantation, their efficacy can be preserved [29]. 

#### 4.2.1. Cell Source

A range of cell types have been utilized for skeletal muscle tissue engineering including SCs, myoblasts, muscle-derived precursor cells (MDPCs), mesenchymal stem cells (MSCs), perivascular stem cells (PSCs), adipose-derived stem cells (ASCs), and induced pluripotent stem cells (iPSCs) [205,206,207]. SCs are a common cell type utilized for tissue engineering because of their essential role as the resident progenitor cell in native tissue regeneration. However, population heterogeneity and purification remain challenges and sources of variability regarding their functional regenerative potential. Additionally, expansion of SCs in vitro results in senescence and loss of proliferative capability, limiting their clinical utility [208]. Thus, there is a tradeoff where increased SC manipulation yields a more purified population but with generally lower regenerative potential [126]. Other muscle-derived cell populations including myoblasts and MDPCs expressing various stem cell markers have also demonstrated regenerative potential, but pose similar challenges regarding cell engraftment, survival, and immunologic rejection [205]. MSCs, including ASCs and PSCs, are also commonly used because they are easily isolated from bone marrow, fat, and umbilical cord tissues, among others [205]. MSCs are pluripotent and give rise to mesodermal tissues including skeletal muscle as well as ectodermal and endodermal tissues. This may permit MSCs to simultaneously participate in peripheral nerve repair and angiogenesis which both play critical roles in skeletal muscle repair. ASCs are a particularly attractive cell type because they are isolated with a simple, high yield procedure and can be expanded quickly, differentiate into myotubes, and evade the host immune system to prevent rejection [206]. These various cell types used for skeletal muscle tissue engineering have been reviewed in detail previously [205,206,207]. Another important concern is whether cellular therapies are derived from autologous or allogeneic sources. Autologous cells present minimal immunogenicity concerns, while allogeneic and xenogeneic cells may require immunosuppressive treatment that potentially limits important native responses that direct skeletal muscle regeneration [209].

#### 4.2.2. Hydrogel-Based Delivery

Hydrogels are one of the most commonly implemented scaffolds for cell delivery-based strategies because cells can be easily mixed into precursor solutions prior to polymerization, and they can be cast directly into the injury, filling irregularly shaped voids. Researchers have used hydrogel scaffolds to deliver myoblasts [210,211], MDPCs [212,213,214], minced muscle grafts [215,216], MSCs [217], ASCs [218,219], and a combination of cell types [220,221]. Beier et al. injected 1 million male primary myoblasts suspended in a fibrin matrix into a female rodent anterior gracilis muscle defect and found male nuclei within host female muscle fibers two weeks after delivery [211]. Other studies delivered MDPCs in fibrin [212,214] and HA [213] hydrogels to partially resected rodent TA muscles, and found that treatment supported reduced fibrosis [212], increased myogenesis, and vascularization [212] and partial restoration of muscle contractile function when delivered progressively via multiple injections over time [214]. Rossi et al. evaluated the delivery of either SCs or MDPCs via a HA hydrogel and found that SCs improved the number of new myofibers and resulted in a significantly higher tetanic force generated compared to delivery of MDPCs alone [213]. Other researchers utilized collagen [215,220] and laminin-111 supplemented HA [216] hydrogels as a vehicle to deliver minced muscle grafts to rodent TA VML injuries [215,216]. Ward et al. found that 50% less minced muscle graft tissue was needed to regain the same functional improvement when delivered in a collagen hydrogel compared to the 100% minced muscle graft [215]. Another study delivering 50% minced muscle grafts with a laminin-111 supplemented HA hydrogel found a 42% improvement in peak tetanic torque production after eight weeks compared to unrepaired limbs. However, this was not a significant improvement compared to delivery of the 50% minced muscle grafts alone. It is not clear if a scaffold is necessary for the delivery of minced muscle grafts, as these cells remain largely contained by their native ECM that remains partially intact. 

Several researchers utilized ASCs for skeletal muscle tissue engineering [218,219]. Aurora et al. delivered human ASCs encapsulated in a composite scaffold of PEGylated platelet free plasma hydrogel with porcine ECM to a rodent TA VML injury [218]. Two weeks after injury the composite scaffold with ASCs resulted in the transplanted cells localized to the injury site and a significantly higher vessel density compared to treatment with acellular composite scaffolds. Another study delivering ASCs in a collagen hydrogel to a similar TA defect found injuries treated with ASC-loaded hydrogels had significantly higher blood flow restoration compared to the hydrogel alone treatment after eight weeks post-injury [219]. It is clear that ASCs encapsulated within hydrogel scaffolds are a promising technology for promoting angiogenesis in VML defects.

Finally, the effect of co-delivery of multiple cell types has also been evaluated [220,221]. One group used composite scaffolds of PLLA/PLGA sponges and fibrin hydrogel to co-deliver ECs and fibroblasts to a defect created in the linea-alba of male nude mice, and 10 days after injury found more neovascularization and significantly higher perfusion than treatment with acellular fibrin hydrogels [221]. While hydrogel delivery systems are easy to deliver and aid in localized cell retention, they are limited due to their weak mechanics and lack of aligned topographical cues necessary to promote functional muscle regeneration.

#### 4.2.3. Decellularized ECM-Based Delivery

Another commonly investigated scaffold for in vivo tissue engineering strategies is decellularized ECM [222,223,224,225,226,227,228,229]. Early work studied the delivery of acellular or autologous myoblast-seeded decellularized muscle ECM to the obliqui abdominis muscle of rats [223]. They found that myoblast-seeded scaffolds demonstrated abundant blood vessels after two months, while acellular scaffolds were completely replaced by scar tissue. Another study used decellularized muscle ECM to deliver male SC-derived myoblasts to a full-thickness abdominal wall defect in female rats and found the persistence of male myoblasts in the implanted patches after nine months [224]. This work suggests that decellularized ECM scaffolds may facilitate the retention of transplanted cells at the injury site. Quarta et al. evaluated whether MDPCs (expressing bioluminescent reporter protein luciferase) encapsulated in decellularized muscle ECM enhanced skeletal muscle regeneration [227]. The researchers discovered that injecting a MDPC suspension directly into ECM scaffolds resulted in inefficient retention within the scaffold, and instead incorporated MDPCs into an ECM hydrogel and microinjected the cell-seeded hydrogel into the bulk decellularized ECM scaffold. MDPCs were delivered alone or in combination with muscle resident cells, a heterogeneous population of fibro-adipogenic progenitors, macrophages, and ECs, to a TA VML injury in mice. ECM scaffolds that co-delivered MDPCs and a muscle resident cell population yielded approximately 40 times higher bioluminescence than scaffolds that delivered MDPCs alone. This suggests that the heterogeneous resident cell population supports MDPC viability within the implanted scaffolds. Additionally, injuries treated with ECM scaffolds co-seeded with MDPCs and muscle resident cells demonstrated active and passive length-tension relationships that were equivalent to control uninjured muscle [228]. Injuries treated with co-seeded scaffolds also exhibited less fibrosis compared to untreated injuries and treatment with scaffolds alone. 

Other researchers utilized decellularized ECM scaffolds to deliver minced muscle autografts [225,226]. An allogeneic decellularized muscle ECM scaffold was used to deliver minced muscle autograft tissue to a rat lower-limb VML injury [225]. After 12 weeks, treatment with minced muscle-loaded ECM scaffolds significantly increased contractile force recovery compared to untreated injury controls. Treated injuries also demonstrated increased MyoD expression and less fibrous scar tissue formation than those left untreated. Goldman et al. created VML defects in the TA of rats and used a UBM ECM scaffold to deliver minced muscle grafts at a dose of 50% of the total defect mass [226]. At eight weeks post-injury, this treatment resulted in a 28.2% significant increase in peak isometric torque compared to the no repair control, whereas treatment with an acellular UBM ECM scaffold did not result in a significant improvement. These studies indicate that decellularized ECM may serve as an effective method to deliver and retain minced muscle tissue within VML defects.

Finally, decellularized ECM has also been used to deliver MSCs [222,229]. Merritt et al. implanted decellularized muscle ECM into a full-thickness defect in the lateral gastrocnemius of Lewis rats [222]. Seven days after implantation, bone marrow-derived MSCs were injected into the implanted ECM. After 42 days an 85% functional recovery was observed in muscles treated with MSC-loaded ECM scaffolds with respect to the contralateral uninjured muscle. However, this functional improvement was not significantly greater than treatment with the acellular ECM scaffold. Muscles treated with MSC-seeded scaffolds had significantly more blood vessels and myofibers than those treated with acellular ECM scaffolds. Another study used human umbilical cord MSCs delivered in a porcine decellularized cardiac ECM because the researchers hypothesized that both components could promote macrophage polarization toward a pro-regenerative M2 phenotype [229]. Rodent TA defects were treated with MSCs, ECM scaffolds, or MSC-loaded ECM scaffolds. Muscle injuries treated with MSC-loaded ECM scaffolds had the highest recorded isometric torque at four and eight weeks post-injury compared to treatment with MSCs alone and ECM scaffolds alone. At two weeks post-implantation, the MSC-loaded ECM treatment group had a significantly higher population of CD206^+^ macrophages, indicative of pro-regenerative M2 polarization. Finally, histological evaluation of treated muscle found treatment with MSC-loaded ECM had higher new muscle fiber regeneration and lower collagen deposition compared to MSCs alone or ECM scaffolds alone. Taken together, varying degrees of success have been demonstrated by using decellularized ECM scaffolds as cell delivery vehicles and may be attributed to differences in ECM sources and decellularization protocols as well as differences in the anatomy and severity of preclinical VML models. 

#### 4.2.4. Microfiber-Based Delivery

While hydrogel, sponge, and decellularized ECM scaffolds have effectively delivered cells to VML injuries, these methods lack aligned topographical cues that have been shown to direct cellular processes that promote improved functional skeletal muscle regeneration. While the use of anisotropic topography has been widely adopted to generate aligned myotubes in vitro, few studies have used aligned scaffolds as a cell delivery vehicle to treat in vivo VML defects [230]. Page et al. investigated the ability of an aligned fibrin microthread scaffold seeded with human MDPCs to restore function in a mouse TA partial resection VML injury [230]. Tissue sections explanted two days and two weeks post-implantation stained with human nuclear antigen revealed that implanted cells had migrated into the host tissue. After 30 days, the percent of collagen was significantly reduced and muscle area was significantly higher in muscles treated with MDPC-seeded fibrin microthreads compared to injuries that received no treatment. After 120 days post-treatment, mice receiving treatment with MDPC-loaded microthreads had significant improvements in tetanic force generation upon electrical stimulation compared to untreated injuries. This study suggests that fibrin microthreads in combination with MDPCs are a promising scaffold for treating VML defects. A recent study performed a meta-analysis of 44 studies that evaluated quantitative functional capacity after treatment of VML injuries by using a random-effects model to evaluate the effect size, which indicates treatment effectiveness [231]. The findings from the Page et al. study had the third highest effect size of the 44 studies that met the inclusion criteria, meaning it resulted in one of the highest improvements in functional capacity. This further validates that an aligned microfiber-based scaffold in conjunction with MDPCs is a promising therapeutic treatment for VML and should be further explored.

#### 4.2.5. Growth Factor-Loaded Scaffolds

As discussed previously (Section 4.1.3.), growth factors are among the most commonly investigated therapeutic molecules for treating skeletal muscle defects due to their role in orchestrating native regeneration [54]. Incorporation of growth factors within scaffolds allows for control over their release kinetics, localized delivery and concentration, and therapeutic effectiveness. Delivering cells within a growth factor-loaded biomaterial scaffold creates a synthetic niche that more accurately mimics ECM and its native signaling molecules. This growth factor-enriched microenvironment may prevent cultured myoblasts from undergoing apoptosis, losing their myogenic potential, and having low transplantation efficiency, which are the major bottlenecks of traditional cell injection-based approaches. Incorporation of growth factors within cell delivery scaffolds may also promote transplanted cell activation, proliferation, migration, and differentiation. Hagiwara et al. implanted FGF2 and green fluorescent protein (GFP)^+^ myoblast-loaded gelatin hydrogel microspheres into a rat thigh muscle injury [232]. After four weeks, muscle injuries treated with FGF2 and myoblast-loaded scaffolds had significantly higher GFP expression compared to the delivery of myoblasts alone or in combination with blank microspheres. Furthermore, this co-treatment strategy yielded significantly higher expression of myogenin and reduced expression of MyoD1, suggesting that this approach promotes myoblast differentiation. 

Studies also investigated the delivery of cells from biomaterial scaffolds containing multiple growth factors [233,234,235,236,237,238]. Complex biomaterials systems that release multiple factors in a spatiotemporal manner mimicking in vivo GF presentation will create a more conducive cellular microenvironment and likely enhance regenerative outcomes [126,192]. Several studies specifically investigated the delivery of IGF-1 and VEGF in tandem [233,234,235]. One study evaluated the co-stimulatory effect of IGF-1 and VEGF by delivering these factors, along with GFP^+^ primary myoblasts, in an alginate scaffold [233]. After myotoxin injury to the murine TA muscle and subsequent hindlimb ischemia, scaffolds were implanted into the injuries. Antibody staining against GFP revealed that after three days of treatment mice implanted with myoblast seeded scaffolds releasing IGF-1/VEGF had a 25.5-fold increase in GFP fibers/mm^2^ over bolus injection of GFP^+^ myoblasts. Scaffolds delivering myoblasts and IGF-1/VEGF also demonstrated the highest recovery of normalized tetanic force compared to all other experimental conditions evaluated. Co-delivery of IGF-1 and VEGF with myoblasts also appeared to have an angiogenic effect on treated muscle injuries, improving blood perfusion after six weeks, compared to those treated with acellular IGF-1/VEGF-loaded scaffolds. Another group used a shape-memory alginate scaffold with IGF-1/VEGF and autologous MDPCs to treat myotoxin and ischemic injuries in TA muscles of mice [234]. Scaffolds delivering IGF-1/VEGF and MDPCs improved transplanted cell engraftment and contractile function after six weeks, with the treatment promoting 90% recovery of the tetanic contractile forces relative to unoperated controls. Another study by Pumberger et al. sought to increase the paracrine signaling of transplanted MSCs by incorporating IGF-1 and VEGF into implanted porous alginate cryogels [235]. They hypothesized that stimulation with IGF-1/VEGF would increase MSC paracrine secretion and induce muscle regeneration without the need for transplanted cells to engraft into the host tissue and directly participate in this process. A crush injury to a rat left soleus muscle was induced, followed by implantation of the MSC-loaded scaffold adjacent to the injured muscle. After 56 days post-injury a significant increase in tetanic contraction and muscle fiber density was observed in injuries treated with MSC-loaded scaffolds with IGF-1/VEGF compared to the blank alginate cryogel control treatment. Incorporation of both IGF-1 and VEGF into biomaterial scaffolds clearly demonstrates their co-stimulatory effect on cell engraftment, angiogenesis, and functional muscle regeneration [233,234,235]. 

Other researchers evaluated FGF2 in tandem with HGF [236] or IGF-1 [237,238]. Hill et al. implanted alginate scaffolds containing SCs, HGF, and FGF2 into murine TA laceration injuries to assess their ability to promote muscle regeneration [236]. Myoblasts delivered on scaffolds releasing HGF/FGF2 had notably higher engraftment into the regenerating muscle compared to myoblasts delivered via bolus injection or acellular HGF/FGF2 loaded scaffolds. Two studies from the Christ lab recently evaluated the co-stimulatory effect of keratin hydrogels loaded with MDPCs, FGF2, and IGF-1 for the treatment of VML defects in rat TA [237] and mouse latissimus dorsi (LD) VML defects [238]. Eight weeks after implantation into TA VML defects, acellular keratin hydrogels incorporated with IGF-1 was the only treatment group that significantly improved functional recovery compared to injuries with no repair [237]. Overall, injuries treated with acellular scaffolds demonstrated greater functional improvements compared to MDPC-seeded scaffolds. A similar result was observed when these scaffolds were implanted in the mouse LD injury [238]. Acellular keratin hydrogels loaded with FGF2/IGF-1 enabled significantly improved recovery of contractile force compared to the same FGF2/IGF-1 loaded scaffold seeded with MDPCs. From these studies, it is clear that FGF2 in combination with HGF or IGF-1 has a co-stimulatory effect in treating muscle injuries, but it remains unclear whether the addition of cells is necessary to further enhance skeletal muscle regeneration. Additional optimization of biomaterial carriers, growth factor incorporation strategies, and cell loading methods are necessary to maximize the functional regenerative potential of in vivo tissue engineering strategies.

#### 4.2.6. Genetically Modified Cells

Another recent strategy to enhance muscle regeneration in VML injuries is the delivery of scaffolds loaded with transfected myoblasts that upregulate myogenic or angiogenic gene expression. While gene therapy is commonly employed, it can be limited by low efficiency and transient transgene expression. These limitations are often due to delivery modality. In addition to utilizing biomaterial-based strategies to more efficiently deliver genetic payloads (reviewed in Section 4.1.3.), researchers investigated the delivery of transfected cells encapsulated within biomaterial scaffolds [239,240,241,242]. Researchers transfected myoblasts [240,241], mesoangioblasts [239], and ASCs [242] to express growth factors involved in muscle regeneration including VEGF [241,242], SDF-1, FGF2 [240], and placental growth factor (PLGF) [239]. Fuoco et al. transduced mesoangioblasts with a lentivector encoding PLGF and encapsulated them within a photopolymerizable PEG-fibrinogen hydrogel [239]. This cell-loaded hydrogel was implanted to replace an 80% ablation of the TA in mice. MyHC^+^ muscle fiber number and size increased with time after injury through six months when muscle fibers appeared fully mature. Grip and tread mill tests revealed that mice whose injuries were treated with transfected myoblasts expressing PLGF demonstrated strength and running resistances almost equivalent to uninjured mice. Another group transfected myoblasts with plasmid vectors carrying human FGF2 cDNA [240]. Myoblasts overexpressing FGF2 were encapsulated in an alginate scaffold and implanted into a crush injury of the rat soleus muscle. After four days post-injury, injuries treated with FGF2 overexpressing myoblasts had a significantly higher number of proliferating cells, lower number of apoptotic cells, and two-fold increase in microvessel formation compared to control animals receiving myoblasts overexpressing luciferase. Despite this, functional analysis at 14 days post-injury did not show a significant increase in muscle force as a result of treatment with FGF2 overexpressing myoblasts. This may be due to evaluating contractile function only two weeks after treatment. Shevchenko et al. transduced human ASCs with recombinant adeno-associated virus encoding human VEGF, encapsulated them in Matrigel, and injected them into the TA, gastrocnemius, and biceps femoris muscles of mice that underwent ischemic injury [242]. They assessed the viability of transplanted cells seven days after intramuscular injection and found the presence of transplanted cells throughout the muscle tissue. After 12 days, injuries treated with VEGF overexpressing ASCs reached 80–90% higher blood perfusion than those treated with non-genetically modified ASCs. Zhou and colleagues transfected myoblasts with VEGF or SDF-1using a lipofectamine transfection reagent [241]. These transfected cells were seeded onto collagen scaffolds and then implanted into rat muscle defects. Implanted conditions include collagen scaffolds and cells transfected with VEGF, SDF-1, or a combination of cells transfected with either VEGF or SDF-1. At two weeks post-injury, microvessel density of the injured tissue was significantly higher in the muscles treated with both VEGF and SDF-1 transfected cells compared to just VEGF, SDF-1, or non-transfected cells. This indicates the synergistic effect of delivering cells transfected with both VEGF and SDF-1. Future work to address challenges including immunogenicity, transfection efficiency, and expression may help expand the use of this promising strategy. Additionally, further optimization of biomaterial carriers to provide a suitable synthetic microenvironment to support cell viability and engraftment will be critical areas of investigation.

### 4.3. In Vitro Strategies: Developing Mature Tissue Constructs Prior to Implantation

While in vivo tissue engineering strategies reviewed in Section 4.2 have resulted in functional regeneration of skeletal muscle defects, this approach has some distinct limitations. Despite reducing the manipulation of cells prior to transplantation and preserving their efficacy, this approach still leaves cells susceptible to low viability, retention, and immune rejection [29,116,117]. In vitro tissue engineering also involves cell delivery, but instead through the development and implantation of a functional tissue engineered muscle graft (TEMG). This is achieved by combining scaffolds, biological factors, and cells, creating cultured constructs in vitro that permit cell differentiation into myofibers. Differentiation is often achieved through a combination of biophysical and biochemical cues as well as mechanical and electrical stimulation. Biomaterial scaffolds with strategic biophysical and biochemical cues create a synthetic microenvironment conducive to cell expansion, differentiation, and ultimately enhanced tissue regeneration. TEMG have the added benefit of being suitable for in vitro disease modeling and drug screening [243,244]. 

While in vitro TEMG have greater functionality prior to implantation than constructs developed through in situ and in vivo tissue engineering strategies, they have several notable limitations. Although TEMGs can generate contractile force, it is often significantly lower than that observed in native muscle tissue [106]. Additionally, oxygen and nutrient diffusion limitations within these constructs often limit their size or require the development of a complex vascular network to support extended cell viability. Prolonged in vitro culture times make TEMG clinical scalability, cost-effectiveness, off-the-shelf availability, and regulatory compliance more challenging than in situ and in vivo tissue engineering approaches. Implanted TEMGs have resulted in varied degrees of skeletal muscle regeneration, which is likely due to vast assortment of scaffold biomaterials, cell types, culture time and conditions, construct sizes, stimulation regimes, custom bioreactors, and preclinical models evaluated [49]. 

#### 4.3.1. Aligned Scaffolds

One of the most facile methods of generating functional TEMG constructs is by utilizing scaffolds with anisotropic surface features to guide aligned myoblast orientation and differentiation. Strategies to generate aligned features are discussed in detail in Section 4.1.2. TEMG constructs have primarily utilized fibrous scaffolds [245,246,247] and aligned pores [170]. The Grayson lab developed hydrogel microfibers by electrospinning co-extruded solutions of fibrinogen and sodium alginate, where the latter acts as a sacrificial structure that when dissolved creates a porous fibrin microfiber [49,245]. They generated TEMGs by seeding these hydrogel microfibers with ASCs [245] or myoblasts [246] and evaluated their ability to regenerate murine VML defects. ASC seeded fibrin microfiber bundles were implanted into a fully resected TA and extensor digitorum longus (EDL) muscle injury [245]. Injuries treated with ASC seeded TEMGs demonstrated minimal fibrosis and significantly more MHC+ cells compared to those treated with acellular fibrin microfiber scaffolds. TEMGs made of fibrin microfibers and myoblasts were also evaluated in a separate study for their ability to participate in skeletal muscle regeneration after a partial ablation of the murine TA [246]. At four weeks post-injury measurements of maximal isometric torque indicated that defects treated with myoblast TEMGs and acellular fibrin microfiber scaffolds demonstrated complete functional recovery, while untreated defects demonstrated a 30% torque deficit compared to uninjured muscles. Another group generated TEMGs by co-culturing myoblasts and ECs on aligned nanofibrillar collagen scaffolds [247]. In vitro, co-cultured aligned nanofibrillar scaffolds generated constructs with longer myotubes, more synchronized contractility, and higher secretion of angiogenic cytokines compared to randomly oriented scaffolds. After nine days of in vitro culture, aligned scaffolds co-seeded with myoblasts and ECs were implanted into a murine partial TA resection injury. Organized myofibers and increased vascular perfusion were observed in muscles treated with aligned TEMGs compared to muscles that received TEMGs with randomly-oriented scaffolds. Finally, Kroehne et al. used a controlled freeze-drying process of collagen gels to create aligned pores 20–50 μm in width [170]. When seeded with myoblasts, the anisotropic pore structure enabled aligned myotube formation in vitro. Upon implantation into a murine TA and EDL resection injury, the TEMG generated force when electrically stimulated. Because of its simplicity and effectiveness, aligned scaffolds continue to be a commonly investigated method of generating TEMGs with aligned myotubes, contractility, and the ability to promote skeletal muscle regeneration in vivo [170,245,246,247]. 

#### 4.3.2. Mechanical and Electrical Stimulation

Another commonly employed method for generating mature and contractile TEMGs is the use of external stimuli such as electrical or mechanical stimulation. These methods create culture conditions that mimic in vivo stimuli and initiate the activation of intracellular signaling pathways involved in skeletal muscle development [248]. Mechanical stimulation regimes mimic uniaxial strains experienced during daily movement and exercise, while electrical stimulation induces muscle contraction and mimics motor neuron electrical synapses. It is well known that immobility (lack of mechanical stimulation) and denervation (lack of electrical stimulation) can cause muscle atrophy [249,250]. Thus, researchers utilized mechanical and electrical stimulation to promote myoblast fusion, alignment, hypertrophy, and contractile TEMGs in vitro [251,252,253,254,255,256,257]. A range of uniaxial mechanical stimulation regimes have been applied to TEMGs, including static and cyclic stretch. Static stretching (or tension culture) provides a single strain at the beginning of a culture period, while cyclic stretching applies periodic strain pulses to TEMGs. Researchers have evaluated different frequencies, amplitudes, and “exercise” durations of cyclic stretch. Similar variables are modified in the development of cyclic electrical stimulation regimes. It is important to note the important role that bioreactors play in the application of these stimulation regimes. Bioreactors are often custom built to control culture parameters as well as provide controlled and precise application of external stimuli.

Electrical stimulation has been used to generate TEMGs that promote myofiber formation when implanted in vivo [251]. Serena et al. seeded MDPCs in a porous collagen sponge and used stainless steel electrodes to apply a cyclic electrical stimulation regime (33 mHz square wave with 70 mM/cm impulses) starting three days after cell seeding [251]. When MDPC seeded collagen constructs were allowed to mature in vitro for seven days, the investigators found that TEMGs that received electrical stimulation had significantly higher expression of MyoD and desmin compared to unstimulated control TEMGs. After one week, TEMGs were implanted into the TA of syngeneic mice. Ten days after treatment, TEMGs were excised and found to contain desmin+ cells and new myofiber formation. While electrical stimulation is a commonly exploited technique for generating mature muscle tissue in vitro, more studies evaluating the ability of these constructs to facilitate functional muscle regeneration upon in vivo implantation must be conducted.

Cyclic mechanical conditioning is also commonly utilized to generate mature TEMGs prior to implantation into VML injuries [252,253,254,255,256,257]. Moon et al. seeded decellularized ECM with MDPCs and used a linear motor to create cyclic uniaxial 10% strain, which stretched the constructs three times/min for the first five minutes of every hour [252]. They found that one week of mechanical preconditioning generated TEMGs with cellular maturation and organization. Preconditioned TEMGs were implanted subcutaneously into the LD of mice. Histological analysis revealed that preconditioned TEMGs demonstrate cellular alignment after one week of implantation. After four weeks, preconditioned TEMGs generated specific force that was 1% of native tissue upon stimulated tetanic contraction, where TEMGs with no preconditioning did not generate detectable contractile force. Machingal et al. used this same mechanical pre-conditioning regime to stimulate their TEMGs of decellularized UBM seeded with MDPCs in a custom bioreactor for one week [253]. TEMGs were then implanted into a 50% resection injury of the LD muscle in mice. After two months, muscles treated with mechanically pre-conditioned TEMGs generated a maximum tetanic force representing 72% of native LD muscle. This is a marked improvement compared to the 50% observed in muscles left untreated or implanted with an acellular UBM scaffold. Corona et al. further investigated these findings by evaluating three versions of the UBM/MDPC TEMG developed by Machingal et al., including (1) a TEMG with short culture time and no pre-conditioning, (2) a TEMG with mechanical pre-conditioning, and (3) a TEMG with pre-conditioning and a second application of MDPCs [254]. Two months after implantation in the same LD model described previously [253] muscle defects that received the TEMG with pre-conditioning and second application of MDPCs had twice the magnitude of functional recovery relative to the other two conditions evaluated [254]. These TEMGs have also been implanted into a more clinically relevant, larger LD defect in an immune competent rat [255]. Two months after injury, ex vivo functional assessment showed that muscle defects implanted with pre-conditioned TEMGs had significantly higher contractile force recovery than those treated with an acellular UBM scaffold or untreated controls. This TEMG with the same cyclic mechanical pre-conditioning protocol has also been evaluated for its ability to stimulate regeneration in murine TA defects, and displayed high variability in functional regeneration [256,257]. Collectively, these studies demonstrate the variability that the VML muscle injury implantation model, anatomical location, and size contribute to observed functional improvements [258]. Future investigations of various scaffold materials and cyclic mechanical stimulation regimes may allow for further TEMG maturation and functionality.

In addition to cyclic mechanical pre-conditioning, many researchers have incorporated static mechanical stimulation to provide uniaxial tension to TEMGs in vitro [244,259,260,261,262]. However, many TEMGs generated with passive tension have not been evaluated in vivo for treating muscle defects, but instead have been developed for in vitro tissue modeling and drug screening [244,260,261,262]. Juhas et al. casted a Matrigel/fibrin matrix seeded with myoblasts into cylindrical polydimethylsiloxane (PDMS) molds with Velcro tabs at either end which acted as gel attachment sites and facilitated uniaxial tension [259]. After two weeks in culture, these TEMGs had a high density of aligned myofibers encased in laminin, and generated twitch and tetanic contractions upon electrical stimulation. Differentiated and undifferentiated TEMGs were both implanted into a dorsal skinfold window chamber in nude mice to evaluate their ability to survive and promote vascularization. Two weeks after implantation, TEMGs were saturated with host vessels, and vessels in pre-differentiated TEMGs appeared to align with myofibers in a manner that mirrors native tissue organization. Functional analysis of explanted TEMGs demonstrated a 3.8-fold increase in specific force from one to two weeks post-implantation. The ability of TEMGs generated with passive tension to treat VML injuries requires future investigation and will allow us to better understand how these constructs may contribute to skeletal muscle regeneration.

#### 4.3.3. Angiogenesis and Innervation

The ability of TEMGs to integrate into host vasculature and become innervated is essential for their survival and ability to promote functional muscle regeneration. Vascularization of these constructs is important because oxygen and nutrient diffusion limitations can inhibit their survival and prohibit the creation of large constructs necessary to treat large-scale VML injuries. To promote angiogenesis, researchers developed pre-vascularized TEMGs in vitro prior to implantation to allow for extended cell viability. This is often achieved through co-culture, where multiple cell types are used to generate TEMGs instead of myoblasts alone. To facilitate the formation of NMJs and prevent denervation-related muscle atrophy of implanted TEMGs, researchers investigated whether pre-forming these structures in TEMGs prior to implantation would accelerate these connections upon in vivo implantation [263]. Additionally, nerve activity has been shown to be essential for skeletal muscle maturation because it provides electrical stimulation and promotes the switch from fast to slow MyHC in regenerating muscle [90]. The development of TEMGs with vasculature and/or NMJs allows for the development of constructs that better mimic native skeletal muscle tissue and increase graft integration and success upon implantation.

Several researchers developed pre-vascularized TEMGs and evaluated their ability to participate in skeletal muscle regeneration in vivo [246,247,264,265,266,267]. Li et al. created TEMGs with collagen hydrogels seeded with adipose-derived microvessels with or without myoblasts [264]. Upon implantation into a full thickness biceps femoris defect, vascularized constructs were unable to prevent fibrosis and did not support muscle regeneration; this may suggest the supportive role that vascularization plays in muscle regeneration. Other researchers utilized co- and tri-cultures to generate vascularized TEMGs [246,247,265,266]. Nakayama et al. seeded myoblasts and ECs on aligned nanofibrillar collagen scaffolds [247]. Vascularized TEMGs yielded twice as many donor-derived myofibers and a significantly higher perfused vascular density compared to acellular scaffolds after implantation in a murine partial TA resection injury. Another group used an electrospun fibrin scaffold co-seeded 1:1 with human ECs and ASCs and cultured the TEMG in vitro for 11 days to form aligned vessels [246]. The researchers implanted these vascularized TEMGs into murine VML defects and found that the implanted human vessels anastomosed with host vasculature and were perfusable. However, little host vessel infiltration into the TEMG was observed, possibly because of the formation of a fibrous capsule around the implanted TEMG. Other researchers evaluated a triculture system of myoblasts, fibroblasts, and ECs to generate TEMGs [265,266]. Koffler et al. seeded myoblasts, fibroblasts, and ECs on decellularized SIS ECM, and found three weeks of culture promoted the formation of vessels with branched networks and multinucleated myofibers [265]. These TEMGs were implanted into a full thickness segment of the abdominal wall of nude mice, and after 14 days exhibited branched vessel networks that permitted higher perfusion than TEMGs of only myoblasts. Similarly, Shandalov et al. created TEMGs by seeding a PLLA/PLGA scaffold with a tri-culture of myoblast, fibroblasts, and ECs that was cultured in vitro for ten days [266]. One week after implantation into a full thickness abdominal wall defect, tri-culture generated TEMGs demonstrated functional vascular perfusion and anastomosis with host vessels. An alternative strategy by Lee et al. developed sheets of myoblasts on a thermosensitive hydrogel transfected with VEGF plasmids [267]. They seeded myoblasts on a Tetronic-tyramine (Tet-TA)-RGD hydrogel. The cells were transfected with VEGF plasmids with poly(b-amino ester) nanoparticles. Transfected cells formed significantly more capillaries in vitro than non-transfected cells. Upon implantation into a mouse ischemia model, muscles treated with transfected cell sheets had significantly enhanced capillary and arteriole density relative to non-transfected cell sheets, indicating the ability of these transfected cells to promote angiogenesis in the injury site. As in vitro tissue engineering strategies continue to be developed, it is necessary to continue the development of strategies to enhance vascularization of these constructs to ensure their survival after transplantation.

Other researchers have sought to form NMJs in vitro to accelerate innervation upon implantation [263]. One study evaluated the ability of agrin to create acetylcholine receptor clusters on TEMGs of myoblast-seeded fibrin hydrogels [263]. Agrin-supplemented TEMGs were implanted subcutaneously into nude rats with the common peroneal nerve embedded within the construct. Agrin-supplemented TEMGs enhanced contacts with host nerves and developed more mature vasculature compared to TEMGs without agrin. However, these constructs did not generate aligned myotubes, likely due to the isotropic nature of the fibrin hydrogel scaffold. Applying agrin supplementation to scaffolds with unidirectional cues may allow for the generation of more mature, innervated TEMGs. The development of TEMGs with a combination of vasculature and NMJs would allow for a more biomimetic construct and one that is likely to have higher survival, anastomosis, and innervation upon implantation.

## 5. Conclusions and Future Directions

This review summarized the current tissue engineering approaches investigated to treat VML defects, with a focus on biomaterials-based strategies. In situ, in vivo, and in vitro tissue engineering strategies offer diverse approaches for treating VML injuries and have demonstrated varied success. The variability of the outcomes is likely a result of the assortment of design factors, including the use of numerous different preclinical defect models and assessment strategies. Many factors impact these models, including the choice of animal, muscle, and the method of generating muscle injury. While mice and rats are the most commonly utilized pre-clinical animal models because of the ease of conducting high throughput studies with standardized injury models, they typically create defects that are orders of magnitude smaller than those seen clinically. More recently, researchers have used larger preclinical animal models including dogs [138] and pigs [268], which generate defects on a more clinically relevant scale [269]. Additionally, muscles in varying anatomical locations have different functions, anatomy, and mechanical loading, which can influence regenerative outcomes [269]. Musculoskeletal injury methods also vary and include myotoxic agents, hindlimb ischemia, and partial or total resection models. While myotoxic agents and hindlimb ischemia can yield significant functional deficits, these injuries retain native ECM, vasculature, and nerve structure. Thus, these injuries have a pathophysiology that is different than that of destructive VML injuries [191]. Finally, researchers utilize a broad range of metrics to evaluate VML regeneration from qualitative histology to quantitative muscle functional analyses. Functional analyses are more useful in assessing regenerative outcomes as a clinically feasible treatment, because VML is characterized by a persistent loss of function. Ultimately, the use of a more standardized preclinical animal model and corresponding functional assessment technique will help to make results more comparable across the field. 

Initially, tissue engineering scaffolds were simple scaffolds that provided mechanical support to injuries or encapsulated cells for more efficient delivery. More recently, they have progressed to more complex materials that recapitulate the ECM native milieu to locally control host cellular functions and guide functional regeneration. To accomplish this, scaffolds strategically combine biophysical and biochemical cues such as mechanics, topography, and biologically active molecules. In the future, scaffolds that concurrently incorporate multiple both biophysical and biochemical cues will more accurately recapitulate native skeletal muscle tissue and likely lead to greater functional improvements. For example, a scaffold that provides mechanical stability, alignment cues, and spatiotemporal control over the presentation of bioactive molecules would provide both biophysical and biochemical cues that promote functional skeletal muscle regeneration. Another important consideration is engineering these scaffolds to promote vascularization and innervation, as both are paramount to normal muscle function and successful regeneration. 

Currently, clinical investigations have been limited to decellularized ECM scaffolds. Ultimately, in situ tissue engineering strategies implanting acellular scaffolds will likely be the first clinically available therapeutic for VML. They have off–the-shelf capabilities and simpler scalability, cost-effectiveness, and path to FDA approval [119]. However, challenges still remain regarding scalability of constructs for large defects and stability of incorporated biologic molecules. Cellular-based strategies have increased complexity compared to acellular strategies. To avoid immune rejection autologous cell sourcing is necessary. This poses significant challenges with manufacturing and maintaining cell plasticity. There is still an unresolved conflict in the literature as to whether cell-based strategies are necessary for treating VML defects. Further research into the mechanisms that govern endogenous regeneration may elucidate mechanisms by which biomaterial-based treatments can successfully in promote functional skeletal muscle in VML injuries.

## Figures and Tables

**Table 1:** Comparison of tissue engineering approaches. Based off Table 1 in [119].

	In Situ	In Vivo	In Vitro
Off-the-shelf availability	Likely	Possible	Not possible
Scalability	Easier	Difficult	Most difficult
Ease of clinical translation	Easier	Complex	Complex
Cost-effectiveness	More	Less	Least
Disease modeling	No	No	Yes
Drug screening modeling	No	No	Yes
